# Comparison of Efficacy of Micropulse Laser Settings for Glaucoma Management

**DOI:** 10.3390/jcm13195753

**Published:** 2024-09-27

**Authors:** Emily Y. Kim, Brooks D. Walker, Nikolas S. Hopkins, Samuel Fowler, Brian M. Jerkins, Elliott M. Kanner, Claire L. Wright

**Affiliations:** Department of Ophthalmology, Hamilton Eye Institute, The University of Tennessee Health Science Center, Memphis, TN 38103, USA; ykim79@uthsc.edu (E.Y.K.); bdwalker14@gmail.com (B.D.W.); nhopkin6@uthsc.edu (N.S.H.); sfowle20@uthsc.edu (S.F.); bjerkins@uthsc.edu (B.M.J.); ekanner@uthsc.edu (E.M.K.)

**Keywords:** glaucoma, intraocular pressure, micropulse laser, trans-scleral cyclophotocoagulation

## Abstract

**Objectives**: This study aims to compare micropulse transscleral cyclophotocoagulation (MP-TSCPC) laser parameters and determine the optimal laser setting. **Methods**: A retrospective study was performed on 351 eyes from patients who underwent MP-TSCPC at four power settings (1500 mW, 2000 mW, 2250 mW, and 2500 mW) from June 2018 to December 2021. The primary measurements of the efficacy of MP-TSCPC were the degree of intraocular pressure (IOP) reduction and the number of glaucoma medication reductions. The rate of hypotony was obtained to assess the safety of MP-TSCPC. **Results:** At 1500, 2000, and 2500 mW, the mean IOP reduction at each visit was statistically significant compared to the baseline, and at 2250 mW, the mean IOP was only significantly different at 18 months (*p* < 0.05). The change in the number of medications with 2000 mW has shown significance at 1 and 3 months from the baseline; with 2500 mW, statistical significance was shown at 3, 6, 12, and 18 months (*p* < 0.05) compared to the baseline. Mean IOP reductions (%) were greater in 2000 mW than in 1500 mW at 1 week, 1 month, and 3 months and were greater in 2500 than in 1500 mW at 1 week (*p* < 0.05). There was no significance for mean IOP reductions at 6, 12, and 18 months across all powers. Only two occurrences of hypotony were reported. **Conclusions**: MP-TSCPC at 1500 mW, 2000 mW, and 2500 mW is a safe and effective treatment for IOP reduction. MP-TSCPC at 2250 mW is safe but may show delayed effectiveness in IOP reduction. In the long term, no one specific power setting was found to be superior for IOP reduction.

## 1. Introduction

Glaucoma is the second leading cause of blindness worldwide after cataracts and poses a significant burden to the world by its irreversible effect. It is estimated that approximately 76 million people were affected by glaucoma in 2020 and that this number will eventually reach 111.8 million by 2040 [1]. Although its pathogenesis is not fully understood, glaucoma is strongly associated with elevated intraocular pressure (IOP), resulting in retinal ganglion cell death and optic nerve damage [2]. Management of IOP is the most well-studied treatment of glaucoma [3,4]. IOP can be reduced with various treatments, including pharmacologic, laser, and/or surgical therapy.

Laser therapy for glaucoma may be performed as first-line or second-line therapy, the latter if treatment with medication or surgery fails to adequately lower the IOP [4]. Traditional transscleral cyclophotocoagulation (TSCPC) is a commonly used therapy that delivers diode laser energy in a continuous manner to the ciliary body, resulting in a reduction in aqueous humor production. While continuous wave (CW) TSCPC effectively lowers IOP, it is associated with significant complications due to thermal damage to the surrounding tissue, including uveitis, loss of visual acuity, hypotony, and phthisis bulbi. Due to these potential complications, CW-TSCPC is often reserved for the treatment of refractory glaucoma [5,6]. Another laser therapy, micropulse transscleral cyclophotocoagulation (MP-TSCPC), has been developed in the last decade and delivers laser energy to the ciliary body using a series of short pulses of laser energy (ON cycle) separated by rest periods (OFF cycle). During the ON cycle, the laser is applied to the ciliary body epithelium, while the adjacent structures have time to cool off and dissipate the thermal energy from non-target tissues during the OFF cycle. By separating the ON and OFF cycles, MP-TSCPC reduces the risk of damaging the surrounding tissues and associated complications while continuing to lower IOP [5,6,7,8].

Numerous studies highlight the clinical use and effectiveness of MP-TSCPC in glaucoma management. In 2022, Gambini et al. [9] published a review article that discusses the multiple published literature works on MP-TSCPC that showed its effectiveness in lowering IOP. However, because these studies used different laser settings, direct comparison was challenging. Within this review article, only one study by Babalola [10] directly compared different spot sizes of MP-TSCPC and discovered that the highest spot size (200 μm) had resulted in a greater IOP decrease. However, the effectiveness and safety of MP-TSCPC are unpredictable when other parameters of the laser are changed.

One retrospective study by Tekeli et al. [7] compared the efficacy and success rate of micropulse laser at two different durations (160 s and 240 s total treatment) and found a similar high success rate in both groups but higher retreatment rate in the group treated for 160 s. Similarly, another retrospective study by Varikuti et al. [11] and two prospective interventional studies by Habash et al. [12] and Preda et al. [13] used comparable durations and laser settings, but the success rate for each study was inconsistent. The variability of these results reflects the difficulty in extrapolating consistently successful micropulse laser settings.

The purpose of this study is to directly compare micropulse laser parameters at a single institution and to determine the optimal settings for maximizing the reduction in both intraocular pressure (IOP) and the number of glaucoma medications while minimizing laser failure and the need for additional glaucoma interventions.

## 2. Materials and Methods

### 2.1. Study Population

A retrospective study was performed on all patients who underwent MP-TSCPC for any type and severity of glaucoma between June 2018 and December 2021 at the Department of Ophthalmology, Hamilton Eye Institute in Memphis, TN, USA. Data were collected from a total of 351 eyes. Preoperative baseline characteristics of patients included age, glaucoma type and stage, preoperative IOP, and the number of glaucoma medications, including both topical and oral IOP-lowering medications. Combination IOP-lowering medications were counted as two separate medications. Intraoperative data consisted of the date of MP-TSCPC, concomitant procedure, and the settings of MP-TSCPC, including laser power (mW), duty cycle (%), number of quadrants treated, and time per quadrant (s). At each follow-up visit, IOP and the number of IOP-lowering medications were recorded, and the need for additional medical or surgical intervention was assessed.

### 2.2. Procedure

All MP-TSCPC procedures were performed at one surgery center under general anesthesia with or without peribulbar or retrobulbar block. One of three glaucoma surgeons (JB, EMK, CLW) performed the procedures at the operating institution. The device used was MicroPulse^®^ laser therapy (IRIDEX, Mountain View, CA, USA), version 2 P3 probe. Laser treatment was typically applied 180 degrees, occasionally 360 degrees, at 31.3% duty cycle. Power settings included 1500 mW, 2000 mW, 2250 mW, and 2500 mW for a duration ranging from 20 to 80 s per quadrant. The power and duration of MP-TSCPC for each patient were determined solely based on the surgeons’ preference and clinical discretion. Post-operative inflammation was managed with topical steroid drops.

### 2.3. Follow-Up

Postoperatively, the patients were examined at six different time intervals: 1 week, 1 month, 3 months, 6 months, 12 months, and 18 months. For patients who did not follow up in the exact time frame, the following time windows were applied: post-operative week (POW) 1 + 1 week, post-operative month (POM) 1 ± 2 weeks, POM3 ± 1 month, POM 6 ± 2 months, POM 12 ± 3 months, and POM 18 ± 4 months. Patients who were lost to follow-up were included in the study, and their IOP and the number of IOP-lowering medications were collected at the corresponding visit when they were present.

### 2.4. Outcome Measures

The efficacy of MP-TSCPC at four different power settings, including 1500 mW, 2000 mW, 2250 mW, and 2500 mW, was measured by the degree of reduction in IOP, the number of medications postoperatively, and the rate of hypotony and retreatment. Hypotony was defined as an IOP < 5 mm Hg for two consecutive post-operative visits. Treatment failure was defined as either IOP < 6 or > 21 mmHg for two consecutive post-operative visits or < 20% reduction in IOP from the baseline for two consecutive follow-up visits after at least one-week follow-up or requiring repeat MP-TSCPC or addition of oral glaucoma medication. Visual acuity was obtained at pre- and post-operative visits, yet it was not collected for analysis. 

### 2.5. Statistical Analysis

Descriptive statistics [mean (SD); n (%)] was completed to report the demographics of patients and characteristics of the glaucoma and their conditions. Kruskal–Wallis rank sum test and Fisher’s exact test were used to derive the *p*-value. A paired *t*-test was used to compare IOPs and the number of medications within each power group and among the power groups. Bonferroni correction was completed to adjust the *p*-value for multiple comparisons. The Kaplan–Meier curve was used to show survival analysis of successful MP-TSCPC at four power settings across the duration of 18-month follow-up. A log-rank test was performed to assess the statistical significance of the survival analysis. Aforementioned statistical tests were completed by using R Project for statistical computing (V4.4.0). One sample *t*-test was used to assess the significance of the difference in the success rate of MP-TSCPC using GraphPad Prism 10. A *p*-value < 0.05 was considered statistically significant.

## 3. Results

### 3.1. Demographics 

A total of 351 eyes underwent MP-TSCPC at the four power settings. The mean age of patients ranged from 63 to 68 years old. There was no significant difference in the age across the power groups (*p* = 0.14). The most common type of glaucoma in all four power groups was primary open-angle glaucoma, making 38–56% of the groups, followed by chronic angle-closure glaucoma in 1500 mW (16%) and combined-mechanism glaucoma in 2000, 2250, and 2500 mW (19–31%). Most of the patients were classified as having severe stage of glaucoma (45–75%) (Table 1).

### 3.2. IOP

**Figure 1:**
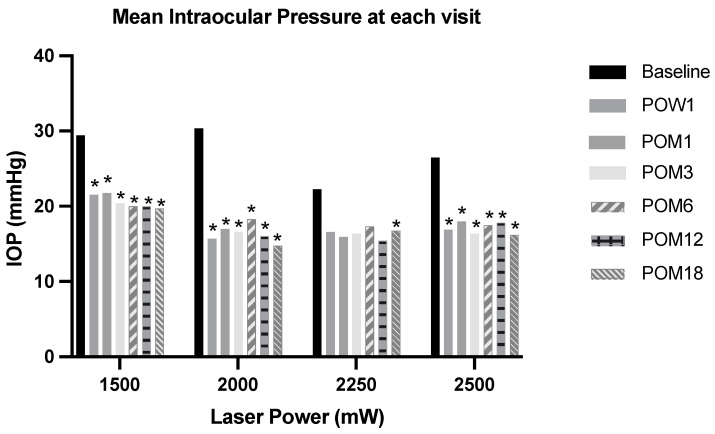
Mean intraocular pressure (IOP) at baseline and each follow-up visit after MP-TSCPC at four power settings. Paired *t*-test was used to assess for statistical significance. * = *p* < 0.05; IOP = intraocular pressure; POW = post-operation week; POM = post-operation month.

**Figure 2:**
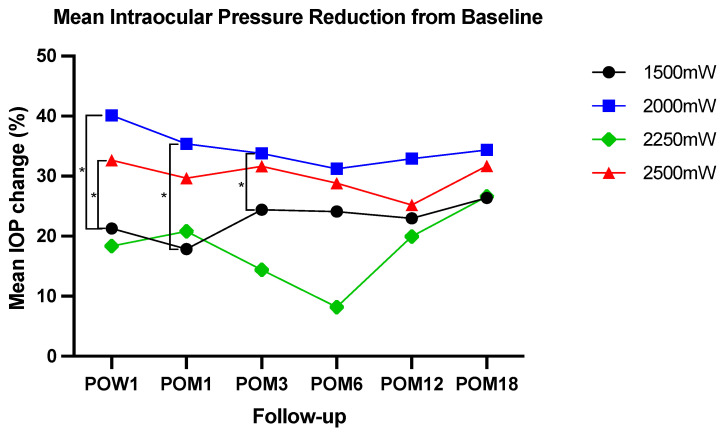
Mean intraocular pressure (IOP) percent reduction from the baseline IOP at each follow-up visit after MP-TSCPC at four power settings. Two sample *t*-test were used to assess for statistical significance between the power groups; * = *p* < 0.05. IOP = intraocular pressure; POW = post-operation week; POM = post-operation month.

The mean IOP at the baseline of each power group in the ascending order was 29 ± 11 mmHg, 30 ± 11 mmHg, 22 ± 8 mmHg, and 26 ± 9 mmHg (*p* < 0.001) (Table 2). The mean IOP after MP-TSCPC at the four power groups during each follow-up visit is shown in Table 2. After MP-TSCPC, there was a significant IOP reduction compared to the baseline at all follow-up visits in 1500 mW, 2000 mW, and 2500 mW. In 2250 mW, the IOP reduction compared to the baseline was significant only at POM18 (Figure 1). The mean IOP percent reductions at follow-ups compared to the baseline IOP in the 1500 mW group are as follows: 1 week, 21.3%; 1 month, 17.9%; 3 months, 24.4%; 6 months, 24.1%; 12 months, 23.0%; and 18 months, 26.4%. Similarly, the mean IOP percent reductions for the 2000 mW group are as follows: 1 week, 40.1%; 1 month, 35.4%; 3 months, 33.8%; 6 months, 31.2%; 12 months, 32.9%; and 18 months, 34.3%. For the 2250 mW group, the mean IOP percent reductions are as follows: 1 week, 18.4%; 1 month, 20.8%; 3 months, 14.4%; 6 months, 8.2%; 12 months, 20.0%; and 18 months, 26.6%. For the 2500 mW group, the mean IOP percent reductions are as follows: 1 week, 32.7%; 1 month, 30.0%; 3 months, 31.6%; 6 months, 28.8%; 12 months, 25.2%; and 18 months, 31.7%. When each power group was compared to one another, 2000 mW showed a significantly greater percent IOP change than 1500 mW at 1 week, 1 month, and 3 months. 2500 mW showed a significantly greater percent IOP change than 1500 mW at 1 week only. No significant IOP reduction differences were seen among the power groups after a three-month follow-up (Figure 2).

### 3.3. Effects on IOP-Lowering Medications

**Figure 3:**
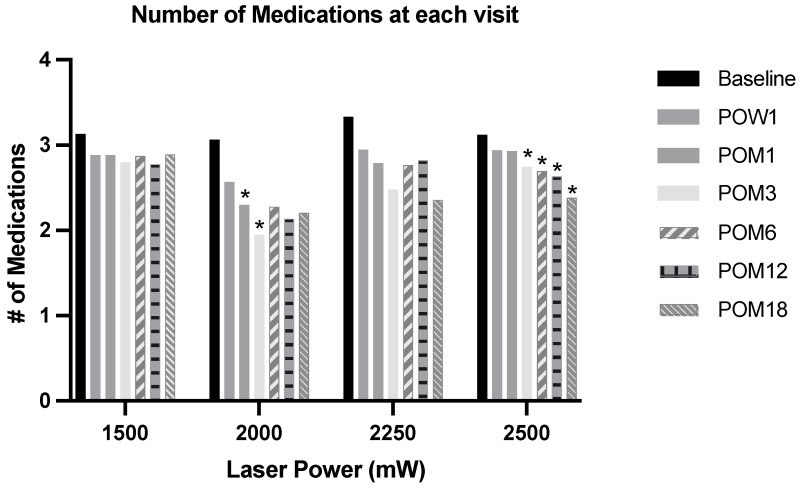
The number of glaucoma medications, including topical and oral formulations, prescribed after MP-TSCPC at the four power settings at each follow-up. Paired *t*-test was used to assess for statistical significance; * = *p* < 0.05. POW = post-operation week; POM = post-operation month.

The baseline number of glaucoma medications of each power group in the ascending order was 3.13 ± 1.17, 3.06 ± 1.39, 3.33 ± 1.15, and 3.12 ± 1.15 (Table 1). The number of glaucoma medications used at follow-up visits after MP-TSCPC at the four power settings is shown in Table 3. Significant differences in the number of glaucoma medications across the four power groups were observed at 3 months and 18 months (*p* < 0.05). When the number of glaucoma medications at each follow-up was compared to those at baseline (3.06 ± 1.39) in the 2000 mW group, there were significant reductions in the number of glaucoma medications at 1 month (2.30 ± 1.41) and 3 months (1.95 ± 1.38) visits. In the 2500 mW group, there were significant reductions in the number of glaucoma medications compared to the baseline (3.12 ± 1.15) at 3 months (2.75 ± 1.04), 6 months (2.69 ± 1.12), 12 months (2.63 ± 1.17), and 18 months (2.38 ± 1.23). No significant effect in the number of glaucoma medications was found in both 1500 mW and 2250 mW groups (Figure 3).

### 3.4. Treatment Success

**Figure 4:**
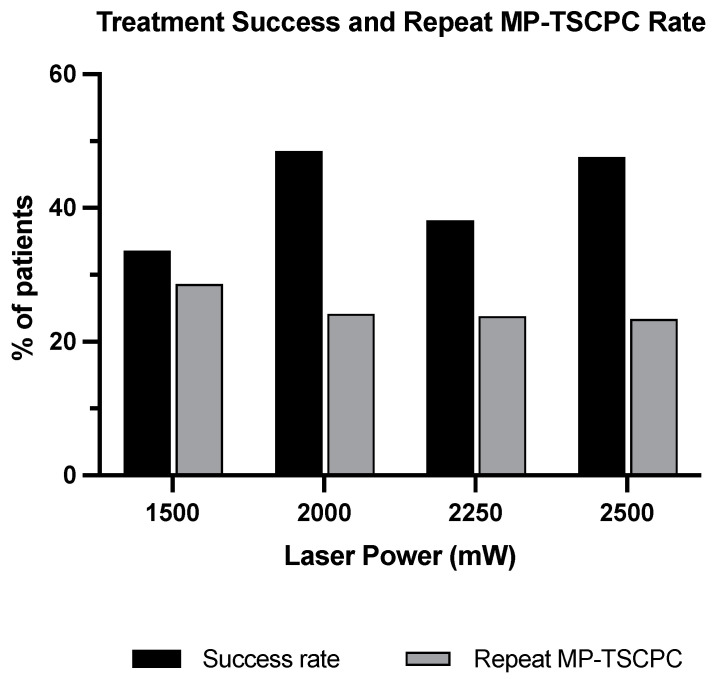
Treatment success and repeat operation rate after MP-TSCPC at the four powers. Treatment was categorized as successful if it did not meet the criteria for treatment failure. Treatment failure is defined if it meets one of the following criteria: (1) IOP < 6 mmHg or > 21 mmHg for two consecutive follow-ups, (2) reduction in IOP from the baseline IOP < 20% for two consecutive follow-up visits after at least 1 week visit, (3) repeat MP-TSCPC, (4) addition of oral glaucoma medication.

**Figure 5:**
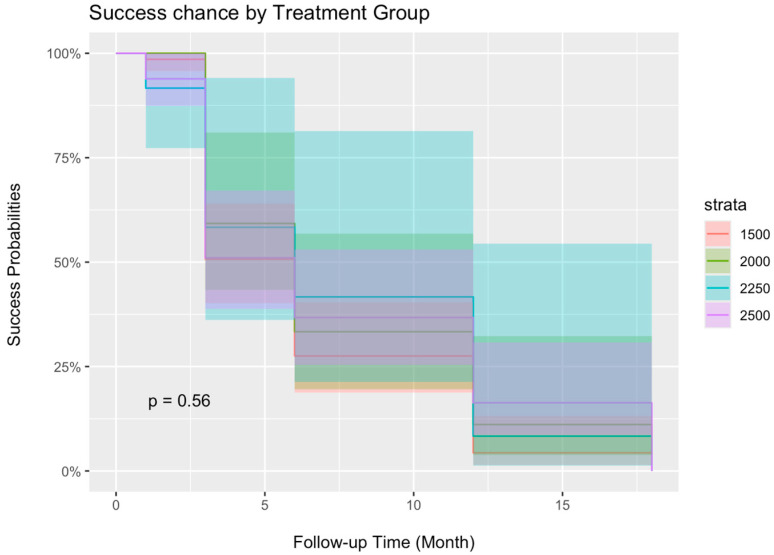
The Kaplan–Meier curve shows the success rate after MP-TSCPC at the four power groups.

In the order of ascending power groups, 1500 mW, 2000 mW, 2250 mW, and 2500 mW, the overall treatment success rates were 33.6%, 48.5%, 38.1%, and 47.6%, and the treatment success rates were significantly different from one another (Figure 4, *p* = 0.0014). The percentage of repeat MP-TSCPC can be found in Table 1 and Figure 4. The Kaplan–Meier curve demonstrates the probability of treatment success after MP-TSCPC using the four power settings at each follow-up visit (Figure 5). There were no significant differences in the survival probabilities of successful treatment among the four power groups (*p* > 0.05).

### 3.5. Rate of Hypotony

Complication of MP-TSCPC was assessed by the rate of hypotony in each power group. In this study, hypotony was defined as IOP < 5 for two consecutive post-operative visits. After MP-TSCPC using 1500 mW, only one patient was found to have hypotony at 12 months and 18 months. Similarly, after TP-TSCPC using 2000 mW, one patient was found with hypotony at 6 months, 12 months, and 18 months. No hypotony was observed in patients after MP-TSCPC using 2250 mW and 2500 mW (Table 1).

## 4. Discussion

In this study, we examined the efficacy of MP-TSCPC determined by the reduction in IOP and the number of anti-glaucoma medications, treatment success rate, retreatment rate, and hypotony rate of MP-TSCPC at four different power settings (1500 mW, 2000 mW, 2250 mW, and 2500 mW) across the six post-operative follow-ups in a patient population with a broad spectrum of severity and types of glaucoma. Furthermore, we aimed to determine which power setting of MP-TSCPC can result in the most effective outcome in IOP reduction.

The greatest mean IOP percent reduction was observed in 2000 mW over the six post-operative visits, followed by 2500 mW, 1500 mW, and 2250 mW. Similarly, at the 18-month follow-up, the 2000 mW (34.3%) group had the greatest IOP percent reduction, followed by 2500 mW (31.7%), 2250 mW (26.6%), and 1500 mW (26.4%). We also found that MP-TSCPC using 2000 mW is more significantly effective at lowering IOP for the first 3 months post-operation than MP-TSCPC using 1500 mW and more significantly effective at lowering IOP 1 week post-operation than 2500 mW. These findings suggest that a power setting of 2000 mW may be more beneficial for patients at lowering IOP in the short term, but no one power setting is superior in the long term. The patients treated with 2250 mW had non-significant IOP changes at all post-operative visits except for the 18-month visit despite similar absolute values of IOP as in the other groups. This finding can be explained by this group’s lower baseline IOP than those in other power groups. Although statistical significances vary across the power groups, the absolute post-operative IOPs are similar in all groups, which suggests that there may be no clear clinical advantages or disadvantages of any of the power settings used in this study. 

The power setting of MP-TSCPC most studied in the current literature is 2000 mW. Many studies have shown the effectiveness of MP-TSCPC using 2000 mW in IOP reduction. Preda et al. and Zemba et al. reported high IOP reductions, 41.8% and 38.3%, respectively, 1 week after the treatment, which is similar to our result (40.1%) [6,13]. Comparable setting to our 2250 mW power group, Al Habash et al. used the power setting of 2200 mW and found a median IOP reduction of 19 mmHg or 52% at 12 months, which is a greater reduction than our finding [12]. The difference in the IOP reductions could be due to our lower baseline IOP, 22 mmHg, than the baseline IOP, 35 mmHg, in their study. Sarrafpour et al. used four different power settings, 2000, 2250, 2400, and 2500 mW, depending on the baseline visual acuity, and found a significant association between the power and IOP reduction. The group treated with 2500 mW showed 57.2% IOP reduction, 51.2% IOP reduction in the group treated with 2250 mW, and 30.1% reduction in the group treated with 2000 mW [14]. In contrast, our results did not reveal an obvious relationship between the power and IOP reduction: the 2000 mW group showed the highest mean IOP change, and the 2250 mW group showed the lowest overall mean IOP change. In Sarrafpour’s study, participants with worse visual acuity treated at higher power had higher baseline IOP (38.3 mmHg in 2500 mW and 21.6 mmHg in 2000 mW), whereas the power of MP-TSCPC in our study was determined based on the surgeon’s discretion, clinical judgments, and personal preference. According to Kaba et al., baseline IOP was a strong predictor of effectiveness [15]. No study was found to compare our group treated with MP-TSCPC using 1500 mW.

In our study, the reduction in the number of IOP-lowering medications was the greatest, with a 1.1 medication drop at a three-month follow-up in the cohort treated with MP-TSCPC at 2000 mW. In this group, the number of medications dropped by 0.9 at an 18-month follow-up. The overall reduction in the number of IOP-lowering medications was the lowest in the group treated with MP-TSCPC at 1500 mW, ranging from 0.2 to 0.4 medication difference. In the cohort treated with MP-TSCPC at 2250 mW, the number of IOP-lowering medications decreased by 0.8 at the 18-month follow-up. In the cohort treated with MP-TSCPC at 2500 mW, the number of medications dropped by 0.7 at an 18-month follow-up. However, our analyses demonstrate that the reduction in glaucoma medications, including both topical and oral formulations, was significant only in 2000 mW at 1 month and 3 months and in 2500 mW at 3-, 6-, 12-, and 18-month follow-ups. The reduction in glaucoma medication after MP-TSCPC was not significant in 1500 mW and 2250 mW. This finding suggests that higher power MP-TSCPC may lead to the use of a lower number of medications post-operatively.

Comparing our results of the number of glaucoma medication reductions to other studies should be performed cautiously due to inconsistent findings in the current literature. Using 2000 mW MP-TSCPC, Preda et al. reported that IOP-lowering medication was decreased by 32% and sustained at 18 months, consistent with our study’s finding (28% reduction) [13]. On the other hand, Tekeli et al. and Zemba et al. reported relatively lower reductions in the number of glaucoma medications at POM 12, 20–26%, and 19%, respectively [6,7], than our study’s finding at 12 months (30% reduction). However, in Zemba et al., the change in the oral IOP-lowering medications was not considered in this analysis. The discrepancies could be attributed to the different MP-TSCPC settings of lower treatment duration per hemisphere despite the same power, 2000 mW, in their studies. Furthermore, Zemba et al. evaluated the effect of MP-TSCPC on patients with neovascular glaucoma only, which is one of the most challenging forms of glaucoma to manage [6]. Using 2200 mW, Al Habash et al. showed a reduction in the number of medications from five to three after the procedure, which sustained until POM 9, then increased to four at POM 12 [12]. Similarly, our findings with MP-TSCPC using 2250 mW showed a fluctuating number of medication changes 3 months after the procedure, which may be related to the demographics of this cohort, as 71% were classified as experiencing severe glaucoma. Overall, the number of glaucoma medications reduced from pre-treatment to POM 18 across all power groups, a similar trend seen in numerous studies.

Rates of MP-TSCPC retreatment varied across powers. Retreatment rates were higher after MP-TSCPC using a lower power setting. The retreatment rate was highest at 28.6% in 1500 mW, followed by 2000 mW (24.2%), 2250 mW (23.8%), and 2500 mW (23.4%). Zemba et al. and Chamard et al. indicated similar findings that 25% and 20% required retreatment after receiving MP-TSCPC at 2000 mW, respectively [6,16]. After MP-TSCPC using higher powers than 2000 mW, Sarrafpour et al. reported a retreatment rate of less than 0.5%, and Fili et al. reported no retreatments in its study group [14,17]. The difference in the retreatment rate seen in the study by Fili et al. and our study may be related to the type of glaucoma of the patients: patients who participated in the study by Fili et al. had either primary open-angle glaucoma (POAG) or pseudoexfoliation glaucoma, where 86% of patients had POAG. Additionally, the criteria for retreatment are highly physician-dependent.

The rate of hypotony, defined by IOP < 5 mmHg for two consecutive follow-ups, was overall very low in all groups: the highest was 1.5% in the cohort treated at 2000 mW, and no hypotony was observed in 2200 mW and 2500 mW groups. In the current literature, variable incidence of hypotony has been reported. Zemba et al. and Tekeli et al. reported hypotony in 8.3% and 0% of patients undergoing MP-TSCPC at 2000 mW, respectively [6,7]. A meta-analysis by Ling et al. supports that many studies that used MP-TSCPC at 2000 mW have reported a low incidence of hypotony (0–9%) [18]. The hypotony seen in our study could be explained by the type of glaucoma those patients with hypotony had. Aquino et al. showed that hypotony has a correlation with neovascular glaucoma [19], and our patients with hypotony were classified with neovascular glaucoma. Similar to our findings, Al Habash et al. and Fili et al. reported no development of hypotony after MP-TSCPC using 2200 mW and 2500 mW, respectively [12,17].

Our treatment success was defined as either IOP ranging from 6 to 21 mmHg or reduction in IOP from baseline > 20% for two consecutive follow-ups after POW 1 or absence of retreatment or no addition of oral glaucoma medication. The treatment success rate was highest in patients treated with MP-TSCPC using 2000 mW (48.5%), followed by 2500 mW (47.6%), 2250 mW (38.1%), and 1500 mW (33.6%). This finding follows a similar pattern as the IOP reduction in the four power groups, indicating that our results are reliable and consistent. Our study demonstrated no significant difference in the treatment success among the four power groups.

Successful treatment criteria widely vary among many studies; thus, the success rates vary greatly. While some studies report success rates of >50% or even as high as 80% [18,19,20], others report lower success rates of <50%, like ours. Valle et al. [21] reported 12-month success rate of 42.9% (success defined as IOP reduction of >20% from the baseline); Souissi et al. [22] reported a success rate of 35% at 12 months (success defined as IOP 6–18 mmHg, >20% IOP reduction, no change in vision, no additional glaucoma medications or glaucoma surgery); and Sanchez et al. [23] reported a success rate of 41% at 6 months (success defined as IOP between 5 and 21 mmHg, IOP > 20% of baseline, no addition of oral glaucoma medications, and no retreatment), which was most similar to our criteria. Our lower rate of success may be explained by a couple of factors. Our definition of success may have been more complicated than other studies. The studies with >50% success rate have similar criteria of IOP range and IOP reduction threshold, yet these studies either neglected to consider the addition of glaucoma medication or laser retreatment as a treatment failure [19,20]. Other reasons may be related to the types of glaucoma, different laser settings, as higher durations are considered to provide a more IOP-reducing effect, and baseline characteristics of patients, including prior surgical history and baseline IOP [18].

Some of the strengths of our study are its long follow-up period of 18 months and the fact that all MP-TSCPC procedures were performed with standardized protocols and techniques by three surgeons at one institution. Additionally, our study included demographics of more than nine different types of glaucoma and vast racial diversity as located in the mid-south region of the United States, whereas many other studies focused on primary open-angle glaucoma and Caucasians. Nevertheless, our study has some limitations. The design of the study was a retrospective chart review rather than a prospective study. Our patient demographics were limited to the southern states of the United States. The sizes of the patient population treated with 2000 mW and 2250 mW MP-TSCPC were relatively small compared to that of the patient population treated with 1500 mW and 2500 mW MP-TSCPC. Lastly, multiple variables, such as duration, type, and severity of glaucoma, may have affected the study’s outcome. Thus, this warrants a subgroup analysis comparing the outcome after exclusively controlling for other variables. 

This study evaluated the MP3 laser using the P3 laser probe. Our institution has since utilized the newly designed probe, which provides a more concentrated and focused beam of photons. Future studies may include safety and efficacy comparisons of our diverse patient population between our original and the new probe. Additionally, a forthcoming analysis of MP-TSCPC involving its effect on visual acuity and visual field would provide additional insight into patient management after MP-TSCPC. 

## 5. Conclusions

In summary, our study reveals that MP-TSCPC performed at powers of 1500 mW, 2000 mW, and 2500 mW are safe and effective treatments for lowering IOP, whereas MP-TSCPC using 2250 mW is safe yet may show delayed effectiveness in IOP reduction until 6 months after the procedure. However, the decrease in IOP-lowering medications was only persistently significant 3 months after MP-TSCPC using 2500 mW. Comparing the four power groups, we found that no one specific power setting of MP-TSCPC is superior for IOP reduction and overall treatment success rate.

## Figures and Tables

**Table 1:** Characteristics of patients with glaucoma who underwent MP-TSCPC at the four power settings.

Characteristics	1500 mW ^1^	2000 mW ^1^	2250 mW ^1^	2500 mW ^1^	*p*-Value ^2^
**Eyes**	n = 140	n = 66	n = 21	n = 124	
**Mean Age (years)**	64 (17)	68 (12)	63 (21)	68 (15)	0.14
**Glaucoma Type**					
Primary open-angle glaucoma	63 (45%)	26 (39%)	8 (38%)	69 (56%)	
Combined-mechanism glaucoma	1 (0.7%)	21 (31.5%)	6 (28.5%)	24 (19%)	
Neovascular glaucoma	18 (13%)	12 (18%)	1 (4.8%)	11 (8.9%)	
Chronic angle-closure glaucoma	23 (16%)	1 (1.5%)	2 (9.5%)	7 (5.6%)	
Secondary glaucoma	9 (6.4%)	0 (0%)	0 (0%)	4 (3.2%)	
Congenital/juvenile glaucoma	9 (6.4%)	1 (1.5%)	1 (4.8%)	2 (1.6%)	
Uveitic glaucoma	7 (5.0%)	1 (1.5%)	2 (9.5%)	0 (0%)	
Traumatic glaucoma	5 (3.6%)	1 (1.5%)	0 (0%)	3 (0%)	
Pseudoexfoliation glaucoma	4 (2.9%)	1 (1.5%)	0 (0%)	1 (0.8%)	
Other	1 (0.7%)	2 (3.0%)	1 (4.8%)	3 (2.4%)	
**Glaucoma Stage**					
Mild	7 (5.0%)	0 (0%)	0 (0%)	5 (4.0%)	
Moderate	21 (15%)	9 (14%)	2 (9.5%)	8 (6.5%)	
Severe	77 (55%)	30 (45%)	15 (71%)	93 (75%)	
Indeterminate	21 (15%)	20 (30%)	0 (0%)	5 (4.0%)	
Absolute	8 (5.7%)	5 (7.6%)	0 (0%)	2 (1.6%)	
Unspecified	6 (4.3%)	2 (3.0%)	4 (19%)	11 (8.9%)	
**Baseline mean IOP (mmHg)**	29 (11)	30 (11)	22 (8)	26 (9)	<0.001
**Baseline number of glaucoma medications**	3.13 (1.17)	3.06 (1.39)	3.33 (1.15)	3.11 (1.15)	>0.9
**Number of Hypotony**	1 (0.7%)	1 (1.5%)	0 (0%)	0 (0%)	
**Number of Repeat MP-TSCPC**	40 (28.6%)	16 (24.2%)	5 (23.8%)	29 (23.4%)	

^1^ Mean (SD); n (%). ^2^ Kruskal–Wallis rank sum test, Fisher’s exact test.

**Table 2:** Mean IOP at each visit after MP-TSCPC at the four power settings.

Characteristics	1500 mW ^1^	2000 mW ^1^	2250 mW ^1^	2500 mW ^1^	*p*-Value ^2^
Baseline mean IOP (mmHg)	29 (11), n = 137	30(11), n = 66	22 (8), n = 21	26 (9), n = 123	<0.001
POW1 mean IOP (mmHg)	22 (9), n = 121	16 (7), n = 60	17 (9), n = 19	17 (7), n = 119	<0.001
POM1 mean IOP (mmHg)	22 (9), n = 111	17(7), n = 59	16 (7), n = 21	18 (7), n = 103	<0.001
POM3 mean IOP (mmHg)	20 (9), n = 99	17 (6), n = 59	16 (7), n = 18	16 (5), n = 97	0.003
POM6 mean IOP (mmHg)	20 (7), n = 95	18 (8), n = 46	16(7), n = 15	18 (7), n = 92	0.004
POM12 mean IOP (mmHg)	20 (8), n = 93	16 (7), n = 36	15 (4), n = 13	18 (8), n = 90	0.012
POM18 mean IOP (mmHg)	20 (10), n = 81	15 (6), n = 24	15 (8), n = 12	16 (6), n = 81	0.006

^1^ Mean (SD). ^2^ Kruskal–Wallis rank sum test, Fisher’s exact test. MP-TSCPC = micropulse transscleral laser cyclophotocoagulation; IOP = intraocular pressure; POW = post-operation week; POM = post-operation month.

**Table 3:** The mean number of glaucoma medications at each visit of the four power groups.

Characteristics	1500 mW ^1^	2000 mW ^1^	2250 mW ^1^	2500 mW ^1^	*p*-Value ^2^
Baseline mean number of glaucoma medication	3.13 (1.17)	3.06 (1.39)	3.33 (1.15)	3.12 (1.15)	>0.9
POW1 mean number of glaucoma medication	2.89 (1.23), n = 122	2.57 (1.41), n = 60	2.95 (1.03), n = 19	2.94 (1.17), n = 121	0.7
POM1 mean number of glaucoma medication	2.88 (1.20), n = 111	2.30 (1.41), n = 60	2.79 (1.31), n = 21	2.93 (1.21), n = 104	0.065
POM3 mean number of glaucoma medication	2.80 (1.32), n = 99	1.95 (1.38), n = 59	2.48 (1.25), n = 21	2.75 (1.04), n = 99	0.001
POM6 mean number of glaucoma medication	2.97 (1.68), n = 96	2.28 (1.32), n = 46	2.76 (1.09), n = 15	2.69 (1.12), n = 95	0.054
POM12 mean number of glaucoma medication	2.78 (1.25), n = 94	2.14 (1.34), n = 37	2.82 (1.07), n = 13	2.63 (1.17), n = 90	0.06
POM18 mean number of glaucoma medication	2.89 (1.20), n = 81	2.21 (1.14), n = 24	2.36 (1.01), n = 12	2.38 (1.23), n = 81	0.008

^1^ Mean (SD). ^2^ Kruskal–Wallis rank sum test, Fisher’s exact test. POW = post-operation week; POM = post-operation month.

## Data Availability

The data will be available upon request to the corresponding author.
